# Highly Efficient Method for Preparing Homogeneous and Stable Colloids Containing Graphene Oxide

**DOI:** 10.1007/s11671-010-9779-7

**Published:** 2010-09-30

**Authors:** Wei Yu, Huaqing Xie, Xinwei Wang, Xiaoping Wang

**Affiliations:** 1 School of Urban Development and Environmental Engineering, Shanghai Second Polytechnic University, 201209 Shanghai, China; 2 Department of Mechanical Engineering, Iowa State University, Ames, IA 50011, USA

**Keywords:** Graphene oxide, Colloid, Phase transfer method, Oleylamine

## Abstract

Phase transfer method has been developed for preparing homogeneous and stable graphene oxide colloids. Graphene oxide nanosheets (GONs) were successfully transferred from water to *n*-octane after modification by oleylamine. Corrugation and scrolling exist dominantly in the modified GONs. GONs were single layered with the maximum solubility in *n*-octane up to 3.82 mg mL^-1^. Oleylamine molecules chemically attach onto the GONs. Compared with traditional strategies, the phase transfer method has the features of simplicity and high efficiency.

## Introduction

Graphene has attracted considerable attention over the past few years due to its exceptional properties [1-3], such as high electrical conductivity, high mechanical strength, and high thermal conductivity. Graphene has exhibited potential applications in microelectronic devices, sensors, biomedicines, and mechanic resonators. Additionally, graphene oxide and graphene can be used as fillers to enhance the mechanical and electrical or thermal transport properties of polymer nanocomposites. The excellent performance of polymer nanocomposites is achieved, not only by using the inherent properties of the nanofiller, but more importantly by optimizing the dispersion, enhancing the compatibility between nanofiller and matrix. The colloidal suspensions containing graphene oxide and graphene are often used to fabricate the composites [4-6]. One of the aims of preparing colloidal suspensions is to enhance the dispersion of nanomaterial in polymer, and the other one is to decrease the toxicity of the composite production [7].

Several approaches have been proposed for the production of aqueous suspensions of graphene oxide and graphene sheets. Exfoliation of graphite oxide either by rapid thermal expansion or by ultrasonic dispersion has been widely adopted to prepare graphene oxide and graphene sheets in bulk. Due to the rich hydrophilic oxygen-containing groups such as carboxyl, hydroxyl, and epoxide, the graphite oxide readily suspends in water and polar organic solvents, such as ethylene glycol, DMF, NMP, and THF at about 0.5 mg mL^-1 ^[8]. In order to enhance the solubility of graphene oxide nanosheets in water, the graphene oxide nanosheets were functionalized with allylamine [9]. The maximum solubility for graphene oxide–allylamine powders in water were determined to be 1.55 mg mL^-1^, which was more than twice of that for bare graphene oxide nanosheets. When *p*-phenyl-SO_3_H groups were introduced into the graphene oxide, the resulting reduced product remained soluble in water and did not aggregate [10]. Phenylene diamine was a good reducing agent and stabilizer for stable graphene colloid, and the as-made graphene could be dispersed well in ethanol, glycol, *N*-methyl-2-pyrrolidone (NMP), but not in *N,N*-dimethylformamide (DMF) [11]. Triblock copolymers (PEO-b-PPOb-PEO) as the solubilizing agent was employed for chemically exfoliated graphite oxide, and graphene formed through in situ reduction by hydrazine [12]. Water-soluble graphene sheets were functionalized by biocompatible poly-l-lysine as a linker through a covalent amide group [13]. KOH could confer a large negative charge through reactions with reactive oxygen-containing groups on the graphene oxide sheets [14], and exfoliated graphite oxide would undergo quickly deoxygenation in strong alkali solutions [15]; therefore, graphene suspension could be prepared by simply heating an exfoliated graphite oxide suspension under strongly alkaline conditions at moderate temperature.

Most of the reported studies are related to the aqueous graphene oxide and graphene suspensions. Organophilic graphene oxide and graphene nanosheets are important for the application in graphene-based composite materials, while only a few papers presented the methods to produce hydrophobic graphene oxide and graphene nanosheets [16]. For example, graphene oxide sheets could be modified by isocyanate [17], which was well dispersed in polar aprotic solvents. The long alkyl chains (such as octadecylamine) [18] were always used as the surface-modified agents and the alkyl-chain-modified graphene sheets that could be dispersed in organic solvents after sonication [19]. Wang et al. [9] reported the synthesis of hydrophobic graphene oxide nanosheets by a solvothermal method, and then they prepared organophilic graphene nanosheets by reacting with octadecylamine [20].

For preparing the suspensions containing graphene or graphene oxide nanosheets (GONs), the traditional strategy is a three-step method. First, graphene or graphene oxide nanosheets are prepared and dried, and then they will be modified. Finally, the functionalized graphene will be dispersed in solvents under stir or ultrasonication. The three-step method is used widely, while there are some drawbacks. First of all, during the synthetic and drying process, GONs have the strong trend to conglomerate due to the large surface area. Second, not all the GONs can be modified, and there is always some sediment under the bottom of suspension. The suspension is not homogeneous, which is not desired for preparing even films and composite materials. Here, we developed a facile phase-transfer method with high efficiency to prepare stable suspensions of graphene oxide in organic solvents. This method is primarily based on the strong interaction between GONs and oleylamine.

## Experimental Section

Natural graphite was purchased from Qingdao Baichuan Graphite Co., LTd. 98% H_2_SO_4_, 30% H_2_O_2_, potassium permanganate (KMnO_4_) and *n*-octane were obtained from Shanghai Chemical Reagents Company. Oleylamine was purchased from Aldrich. All other reagents were used without further purification.

**Scheme 1:**
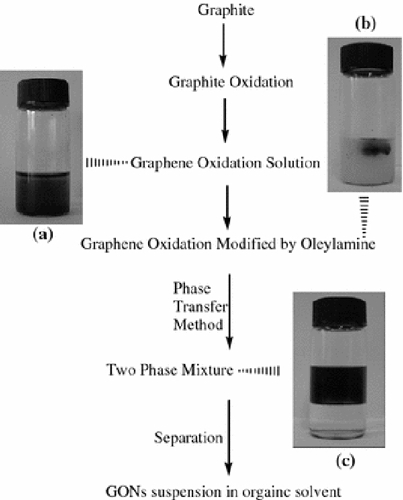
Synthesis process of suspensions containing GONs in organic solvents.

The synthesis process of suspensions of graphene oxide in organic solvents is presented in Scheme 1. At first, nature graphite powder was oxidized to graphite oxide using a modified Hummers method [21,22]. The obtained graphite oxide (0.1 g) was dispersed in 20 mL de-ionized water and exfoliated to generate GONs by ultrasonication for 12 h. The result was a homogeneous dark brown solution (Scheme 1a) of dispersed GONs. Then 0.5 g oleylamine was added in the colloidal suspension. Due to the strong interaction between oleylamine and GONs, oleylamine was absorbed on the surface of GONs, and GONs became hydrophobic GONs-OA, and floated in the water (Scheme 1b). Then 20 mL of *n*-octane was added into the mixture. The phase transfer process occurred spontaneously, and there was a distinct phase interface between the aqueous and octane in 1 day (Scheme 1c). After removing the aqueous phase using a pipette, the stable suspensions of graphene oxide in octane was obtained.

The size and morphology of the GONs were examined by using transmission electron microscopy (TEM, JEOL 2100F). The TEM samples were prepared by dropping the diluted colloid onto a carbon film mesh supported on a copper grid and drying it in air. Atomic force microscopic (AFM) images were taken on a MultiTask AutoProbe CP/MT Scanning Probe Microscope (Veeco Instruments, Woodbury, NY). Imaging was done in tapping mode using a V-shaped 'Ultralever' probe B and nominal tip radius 10 nm. The diluted GONs colloid was dropped onto a freshly cleaved mica. After the mica was dry, the GONs-OA were imaged. FT-IR spectra were recorded with a Bruker Equinox V70 FT-IR spectrometer in dry KBr pullet in the range of 400–4,000 cm^-1^. A thermogravimetric analyzer (TG-DTG, Netzsch STA 449C) was used for thermogravimetric analysis and calculation of the decomposition activation energy (sample mass: about 15.0 mg; atmosphere, flowing dry nitrogen). The UV–Vis spectra of the octane suspensions containing GONs were measured on a Shimadzu UV2550 UV–Vis spectrometer. In order to determine the concentration of the suspension, the calibration line was constructed by measuring UV–Vis spectra absorption intensities of the suspensions at different concentrations (0.1–0.5 mg mL^-1^). The standard solution with 0.5 mg mL^-1^ was prepared through the following process. At first, 20 mL of 0.5 mg mL^-1^ aqueous solution containing GONs was prepared, then 50 mg oleylamine was added, after that GONs-OA would be transferred to 20 mL octane, and the resulting was the octane suspension containing GONs with the concentration 0.5 mg mL^-1^. To determine the maximum solubility of the suspension, the supernatant solution of the saturated suspension was diluted several times for the measurement of UV–Vis spectra until the absorbance fitting in the range of calibration line. Based on the calibration line, the concentration of the diluted solution could be determined. Finally, the maximum solubility could be obtained based on the times of the dilution.

## Results and Discussions

**Scheme 2:**
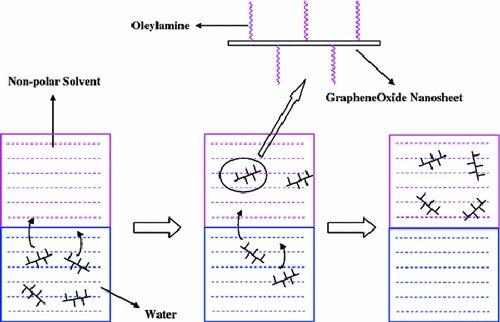
Schematic illustration of the phase transfer process.

Oleylamine is a typical surface modification agent, and it is often used to prepare enhanced hydrophobic nanomaterials [23]. Graphene oxide with rich oxygen-containing groups is expected to be surface-modified by oleylamine easily, and the experimental results validate the idea. The synthesis process of suspensions containing GONs in organic solvents was presented in Scheme 1. At first, the aqueous solution containing GONs was prepared, and then the GONs modified by oleylamine (GONs-OA) in water would be transferred to the organic phase *n*-octane. The phase transfer process of preparing organosol was simple. The GONs modified by oleylamine could be transferred from aqueous solution to organic solvent easily with high efficiency. Almost all the GONs could be transferred to octane (Scheme 2). The prepared colloids were homogeneous, and they remained stable for more than 6 months without any sediment.

**Figure 1:**
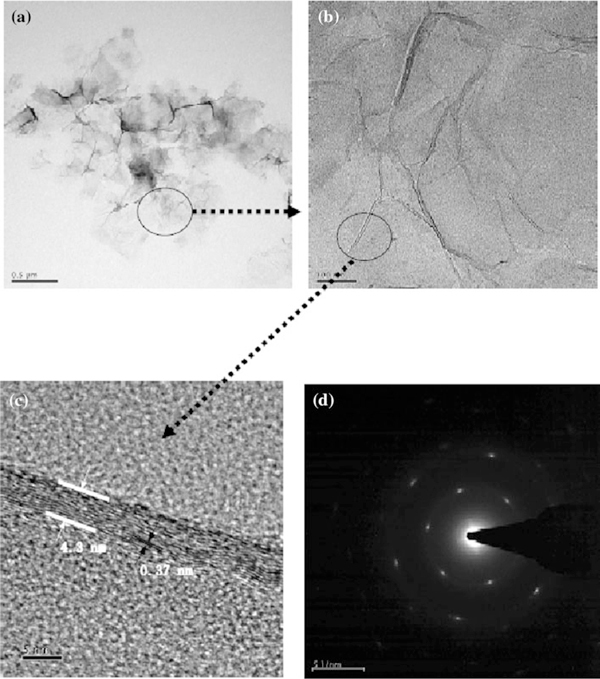
**a Low magnification of TEM view of GONs-OA, b rippled GONs-OA with waves, c HRTEM image of as-prepared GONs-OA and d corresponding to the selected area electron diffraction pattern (SAED)**.

**Figure 2:**
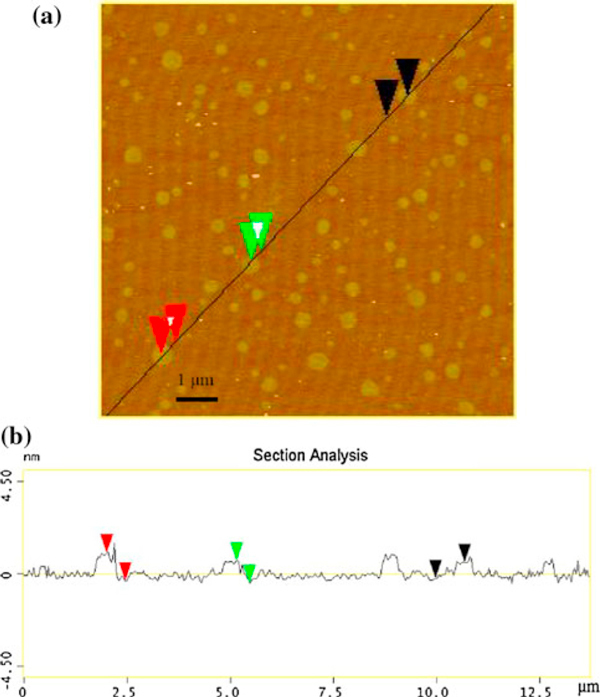
**Tapping mode AFM image of GONs-OA (a) and height profiles in selected location (b)**.

Figure 1 shows the typical TEM images of GONs-OA. The sizes of GONs-OA were in the range of 300–800 nm. The 2-D membrane structure will be thermodynamically stable via bending; therefore, graphene always exists in the state of corrugation and scrolling [9]. Figure 1b demonstrated the corrugation clearly, and the thickness of the corrugation was 4.3 nm (Figure 1c). The HRTEM image of the corrugated GONs-OA illustrated the graphitic lattice clearly, and the interplanar distance was measured to be 0.37 nm, corresponding to the spacing of the (002) plane. The diffraction dots were indexed to the hexagonal graphite crystal structure. The cross-sectional view of the typical AFM image of the GONs-OA (Figure 2) indicated that the average thickness of GONs-OA was about 1.3 nm, and the thickness of GONs-OA was very even. In our opinion, almost all the sheets were single-layer graphene oxide. The thickness was somewhat larger than that of single-layer GONs (0.8–1.0 nm) [24], attributed to the presence of oleylamine absorbed on both sides of GONs. This is very important because most of the fascinating properties of graphene-based composites are mainly related with their layer numbers of graphene.

**Figure 3:**
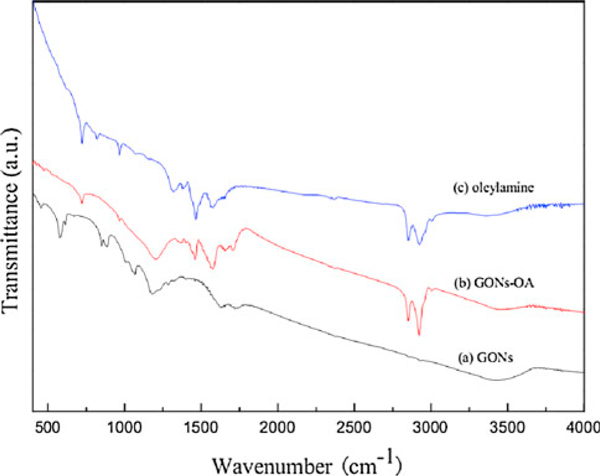
**FT-IR spectra of GONs (a), GONs-OA (b), and oleylamine (c)**.

The phase transfer method is based on the strong interaction between GONs and oleylamine, which is confirmed by the FT-IR spectral analysis. Figure 3a shows the typical absorption bands of GONs, such as O–H stretching vibration (3,400 cm^-1^), C=O stretching of carboxyl groups situated at edges of GONs sheets (1,730 cm^-1^), epoxide groups and skeletal ring vibrations (1,630 cm^-1^) and the stretching vibration of C–O–C (1,184 cm^-1^) [16]. Figure 3b shows the FT-IR spectrum of GONs-OA powders. The asymmetric bands of the alkyl group at 2,850 and 2,921 cm^-1^ in GONs-OA correspond to the C–H stretching vibrations in oleylamine. The amine group (N–H) is also observed with a band 1,458 cm^-1^ for GONs-OA, which corresponds to 1,465 cm^-1^ in the FT-IR spectrum of oleylamine (Figure 3c). Compared with GONs, GONs-OA do not show the strong absorption at 1,730 cm^-1^, and two new bands appear at 1,656 and 1,438 cm^-1^, corresponding to *ν*_as_(COO^-^) and *ν*_s_(COO^-^), respectively. The facts illustrate that the –COOH groups of GONs is ionized to –COO^-^, and it clearly confirms that oleylamine is attached to GONs through chemical absorption.

Kahn prepared soluble oxidized single-walled carbon nanotubes (SWNT) in organic and aqueous solvents through derivatization using 2-aminomethyl-18-crown-6 ether (CE) [25]. They proposed that SWNT and CE formed SWNT-CE adduct, arising from a twitter ionic interaction between a protonated amine on CE and an oxy-anion from a carboxylic acid group, creating a COO^-^NH_3_^+^ ionic bond. Li et al. investigated the extraction of maleic acid using trioctylamine, and they found the amine formed 1:1 complexation with maleic acid through ion pairing [26]. Zhou et al. reported the crystal structure of the ethylamine salt of 2-(4-isobutylphenyl) propionic acid [27], and the result verified the above proposition. In the paper, there is a strong interaction between oleylamine and GONs, and the interaction is very similar to that between SWNT and CE.

**Figure 4:**
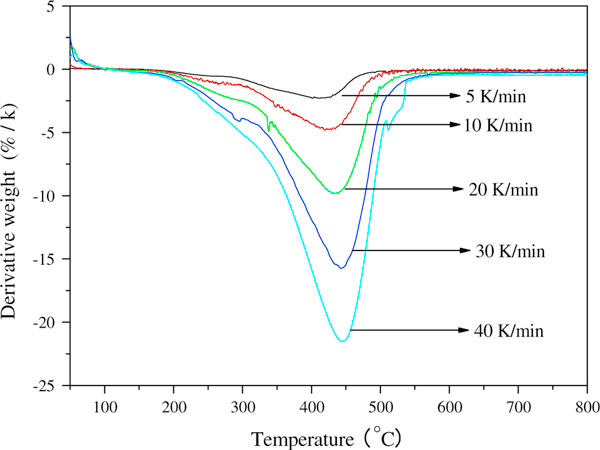
**Derivative thermogravimetric plots of GONs-OA at different heating rate**.

**Figure 5:**
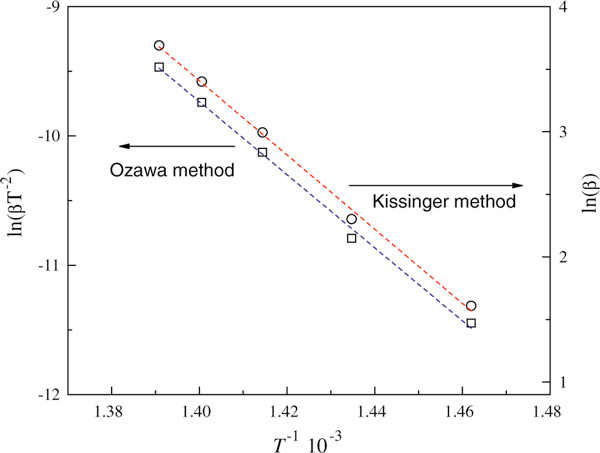
**Linearization curves of Ozawa method and Kissinger method**.

In order to further investigate the interaction of GONs and oleylamine, the decomposition activation energy of GONs-OA was investigated through the thermo-gravimetric analysis under nitrogen atmosphere with the heating rate 5, 10, 20, 30, and 40 K min^-1^. The derivative thermogravimetric plots of GONs-OA at different heating rate are shown in Figure 4. The linearization equations of Ozawa method and Kissinger method are *y* = -29.64817 × 10^3^*x* + 44.91489 (*R*^2^ = 0.997) and *y* = -28.24605 × 10^3^*x* + 29.80937 (*R*^2^ = 0.997), respectively (Figure 5). The decomposition activation energy calculated through Ozawa and Kissinger methods are 234.31 and 234.84 kJ mol^-1^, respectively. The decomposition activation energy is large, also demonstrating the strong interaction between oleylamine and GONs.

**Figure 6:**
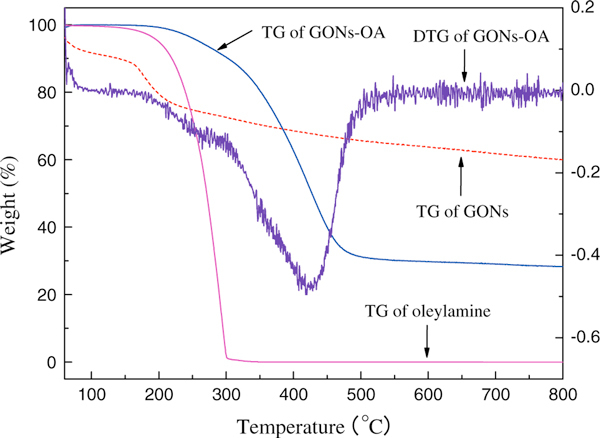
**Thermogravimetric curves of oleylamine, GONs-OA, and GONs**.

Figure 6 shows the thermogravimetric curves of oleylamine, GONs-OA, and GONs. Graphene oxide is thermally unstable, and the thermo-gravimetric analysis of GONs has been investigated in detail by some references [28]. The initial weight loss of GONs below 100 °C is ascribed to the elimination of adsorbed water. The major mass loss is in the range of 150–220 °C, presumably due to pyrolysis of the labile oxygen-containing functional groups, yielding CO, CO_2_, and steam. For the TG curve of pure oleylamine, there is an obvious weight loss process with an onset temperature 145 °C, corresponding to the boiling point of oleylamine. GONs-OA displays different thermal behaviors. For GONs-OA, below 150 °C there is no weight loss. Because of the hydrophobic property of GONs-OA, polar molecules will not be absorbed on the surface. The onset temperature of GONs-OA is 178 °C, and the main mass loss is in the range of 280–520 °C with the peak at 425 °C. The process is due to the decomposition of the organic functional groups and further carbonization of the graphene backbone. The weight loss for GONs-OA from 50 to 800 °C is 71.7%, mainly due to the absorbed oleylamine molecules. The left 28.3% is graphene oxide. Through a crude calculation, we can obtain the molar ratio of graphene carbon and oleylamine, it is 8.8:1. Because GONs-OA were washed by hot ethanol for 5 times, oleylamine molecules would be absorbed on the surface by monolayer absorption. Therefore, it is estimated that about 11.4% carbon atoms of GONs is absorbed by oleylamine molecules.

**Figure 7:**
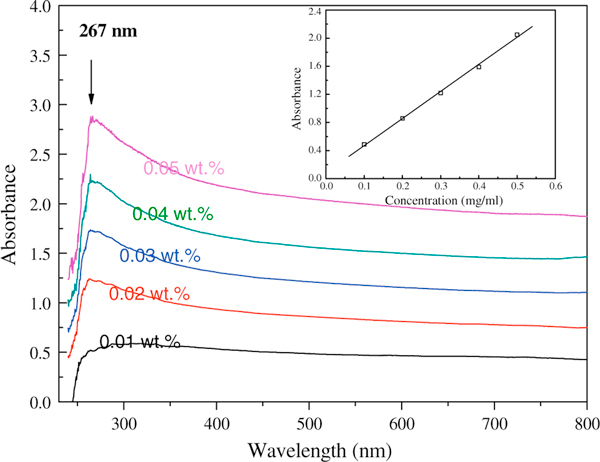
**UV–Vis spectra for GONs-OA suspensions in *n*-octane at different weight concentration**. The *insert* shows the linear relationship between the UV–Vis absorbance and the concentration at the wavelength of 500 nm.

Figure 7 shows UV–Vis absorption spectra of GONs-OA suspensions in *n*-octane at different weight concentration. Due to the absorbed oleylamine molecules, GONs-OA suspensions present the characteristic absorption band at 267 nm, assigned to the C=C of oleylamine. The band of GONs-OA at 267 nm is so strong as to shadow the typical absorption peak at 230 nm of GONs [9]. According to the Beer's law, there is a linear relationship between the absorbance and concentration of the solution. The solubility of GONs-OA powders in octane could be quantitatively characterized by UV–Vis spectroscopy. Due to the existence of free oleylamine in the suspension, we would not obtain the accurate concentration of GONs-OA based on the peak at 267 nm. Oleylamine has no absorption at 400–800 nm, so we constructed the calibration line by measuring UV–Vis spectra of GONs-OA suspensions in *n*-octane at different concentration at the wavelength of 500 nm. The inserted figure in Figure 7 shows the relationship between the absorbance at 500 nm and the concentration of GONs-OA. Based on the calibration line, the maximum solubility of GONs-OA in octane solvent was calculated to be 3.82 mg mL^-1^. Wang et al. used the same method to obtain the solubility of hydrophobic graphene oxide nanosheets prepared via functionalizing with phenylisocynate through a solvothermal synthesis process, and the maximum solubility of GONs in DMF solution was determined to be 0.56 mg mL^-1 ^[9]. Comparing with the solvothermal synthesis process and the three-step method, phase transfer method is very easy and effective for preparing homogeneous and stable suspensions containing graphene oxide in organic solvents.

## Conclusion

Optimizing the dispersion of graphene oxide in non-polar solvent is vital for the application and production of graphene-based composites. Synthetic methods for the colloidal suspensions containing graphene and graphene oxide are one of research hotspots. In this paper, the phase transfer method was applied to prepare homogeneous and stable suspensions containing GONs in non-polar solvents. Compared with the traditional three-step method, phase transfer strategy is very simple. Due to the strong interaction between oleylamine and GONs, almost all the GONs could be transferred to the organic phase with high efficiency. The size of GONs-OA was in the range of 300–800 nm, and GONs-OA always existed in the state of corrugation and scrolling. Almost all the sheets were single-layer graphene oxide, and the thickness of GONs-OA was uniform. Due to the presence of oleylamine absorbed on both sides of the GONs, the average thickness of GONs-OA was about 1.3 nm. The FT-IR spectra clearly confirmed that oleylamine molecules were attached to GONs through chemical absorption. The thermo-gravimetric analysis of GONs-OA illustrated that about 11.4% carbon atoms were absorbed by oleylamine. The decomposition activation energy calculated through Ozawa and Kissinger method were 234.31 and 234.84 kJ mol^-1^, respectively, indicating the strong interaction between oleylamine and GONs. The solubility of GONs-OA in octane was quantitatively characterized by UV–Vis spectroscopy, and the maximum solubility of GONs-OA in octane solvent was up to 3.82 mg mL^-1^.
